# β cell acetate production and release are negligible

**DOI:** 10.1080/19382014.2024.2339558

**Published:** 2024-04-12

**Authors:** Kai Xu, Chioma Nnyamah, Nupur Pandya, Nadia Sweis, Irene Corona-Avila, Medha Priyadarshini, Barton Wicksteed, Brian T. Layden

**Affiliations:** a Division of Endocrinology, Diabetes and Metabolism, University of Illinois at Chicago, Chicago, IL, USA; b Jesse Brown VA Medical Center, Chicago, IL, USA

**Keywords:** Acetate, FFA2 and FFA3, Insulin secretion, liquid chromatography mass spectrometry, pancreatic β cells

## Abstract

**Background:**

Studies suggest that short chain fatty acids (SCFAs), which are primarily produced from fermentation of fiber, regulate insulin secretion through free fatty acid receptors 2 and 3 (FFA2 and FFA3). As these are G-protein coupled receptors (GPCRs), they have potential therapeutic value as targets for treating type 2 diabetes (T2D). The exact mechanism by which these receptors regulate insulin secretion and other aspects of pancreatic β cell function is unclear. It has been reported that glucose-dependent release of acetate from pancreatic β cells negatively regulates glucose stimulated insulin secretion. While these data raise the possibility of acetate’s potential autocrine action on these receptors, these findings have not been independently confirmed, and multiple concerns exist with this observation, particularly the lack of specificity and precision of the acetate detection methodology used.

**Methods:**

Using Min6 cells and mouse islets, we assessed acetate and pyruvate production and secretion in response to different glucose concentrations, via liquid chromatography mass spectrometry.

**Results:**

Using Min6 cells and mouse islets, we showed that both intracellular pyruvate and acetate increased with high glucose conditions; however, intracellular acetate level increased only slightly and exclusively in Min6 cells but not in the islets. Further, extracellular acetate levels were not affected by the concentration of glucose in the incubation medium of either Min6 cells or islets.

**Conclusions:**

Our findings do not substantiate the glucose-dependent release of acetate from pancreatic β cells, and therefore, invalidate the possibility of an autocrine inhibitory effect on glucose stimulated insulin secretion.

## GRAPHICAL ABSTRACT

**Figure:**
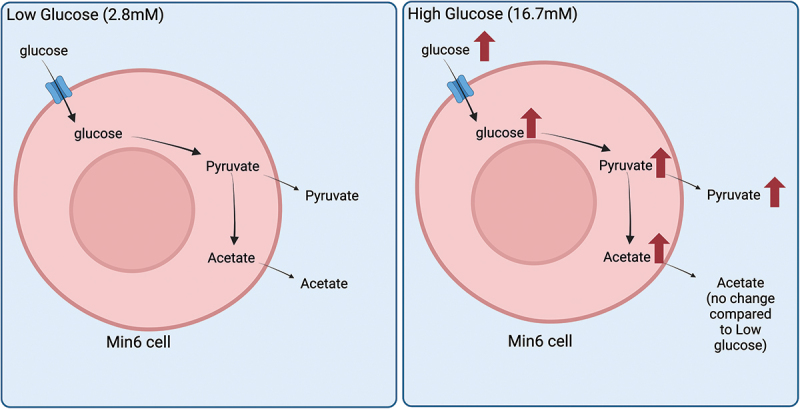

## Introduction

In the pathogenesis of T2D, pancreatic β cell dysfunction occurs prior to the development of overt T2D symptoms.^1,2^ Thus, the preservation of β cell function is a key aspect in the prevention of development and progression of T2D. Insulin secretion, the primary function of β cells, is dysregulated in T2D.^3,4^ For this reason, extensive research has examined the mechanisms underlying insulin secretion, and this is a key therapeutic target against T2D. The widely studied insulin secretory pathway begins with glucose feeding into glycolysis and subsequently, into the tricarboxylic acid (TCA) cycle for ATP production. The resultant increase in the intracellular ATP:ADP ratio leads to the closure of ATP-sensitive potassium channels and recruitment of extracellular calcium, followed by endoplasmic reticulum calcium release, eventually leading to insulin secretion.^1^ Drugs that target components of this established pathway (e.g., sulfonylureas that block the potassium channels) are widely used as T2D medications.^5^

Recently, attention has been drawn to additional metabolic pathways as potential targets for regulating insulin secretion. Researchers have shown that the anaplerotic metabolism of pyruvate, producing reduced equivalents (e.g., malate pyruvate shuttle and citric pyruvate shuttle, which produce NADPH), is positively correlated with glucose-stimulated insulin secretion (GSIS).^6,7^ The pentose phosphate pathway produces intermediates, such as adenylosuccinate, that have been shown to stimulate insulin secretion.^8^ Additionally, amino acids such as glutamine and leucine can also potentiate insulin secretion in the presence or absence of glucose.^9–12^ Acute incubation of L-alanine in addition to glucose can accelerate the TCA cycle in the β cells leading to the stimulation of insulin secretion.^13,14^

Fatty acids have also been shown to affect insulin secretion. While *ex vivo* stimulation of islets with fatty acids alone cannot elicit insulin secretory response, long-chain fatty acids are able to act through GPR40 (a G protein-coupled receptor) to increase insulin secretion in the presence of glucose.^15,16^ Recently, new evidence has shown that SCFAs also regulate insulin release through similar pathways. FFA2 and FFA3, which are the GPCRs from the same family as GPR40, are known to be expressed in pancreatic β cells.^17–21^ Their ability to regulate insulin secretion upon interaction with SCFAs such as acetate, propionate, and butyrate *in vitro* has been reported by multiple groups. As SCFAs are derived from gut microbial activity, reports on their role in insulin secretion have evoked special interest. These reports have facilitated our initial understanding of SCFAs-mediated modulation of insulin secretion. For example, acetate at millimolar concentrations has been shown to potentiate islet insulin secretion in both static GSIS and perifusion studies (19, 23, 24). Importantly, one report observed that β cells could release acetate into the extracellular environment in a glucose-dependent manner,^20^ suggesting autocrine regulation of acetate-mediated insulin secretion through FFA2 and FFA3. One caveat, however, is that the methodology used for quantification of β cell released acetate, which is a food-grade: enzyme-based colorimetric assay. This assay, like most commercially available assays for acetate, is imprecise due to a lack of specificity and sensitivity (detection limit at 2.5 mg/L = 41.6 µM, limit of quantification 4.5 mg/L = 75 µM), where acetate concentration measured were well below assay limit in these experiments. Additionally, acetate efflux requires monocarboxylate transporters (MCTs), specifically MCT1–4, which are not expressed in pancreatic β cells.^22^ Thus, it is necessary to understand in detail how pancreatic β cells release acetate. An additional concern about this approach is that SCFAs are volatile molecules and need to be derivatized to remain stable during the methods used to measure them. Currently, liquid chromatography-mass spectrometry (LC-MS) together with chemical derivation to address SCFA volatility is the gold standard to measure acetate with accuracy and sensitivity in biological samples.^23–25^ As a result, it is critical to reevaluate this autocrine production/release of acetate. Through rigorous approaches and precise LC-MS measurements, in the present study, we observed that both intracellular pyruvate and acetate increased with high glucose conditions; however, intracellular acetate levels increased only slightly and exclusively in Min6 cells but not in the mouse islets. Further, extracellular acetate levels were not affected by the concentration of extracellular glucose in the incubation medium of either Min6 cells or mouse islets. Overall, we showed that β cells do not release acetate in a glucose-dependent manner, and therefore these data demonstrate the unlikely possibility of an autocrine effect of acetate on insulin secretion.

## Materials and methods

### Mouse islet isolation and culture

CD-1 male mice (Charles River Labs, MA, USA) were housed in a temperature and humidity-controlled specific pathogen-free barrier facility with ad lib access to food (Envigo-7912, IN, USA), water, and 12 hours of dark and light cycle until used for islet isolation. All procedures were approved by The University of Illinois at Chicago Animal Care Committee. Islets from 10-week-old CD-1 male mice were isolated by collagenase digestion as previously described.^19^ After isolation, mouse islets were left to recover in RPMI 1640 medium (Gibco, MA, USA) supplemented with 10% FBS (Sigma Aldrich, MO, USA) 1% L-glutamine (Gibco) and 1% penicillin/streptomycin (Gibco) overnight at 37°C in 5% CO_2_, 95% air environment.

### Min6 cell line culture

Min6 cells were cultured in DMEM high glucose with sodium pyruvate medium (Gibco) supplemented with 15% FBS (Sigma Aldrich), 1% L-glutamine (Gibco), 1% penicillin/streptomycin (Gibco), and 0.1% β-mercaptoethanol (Gibco) in 5% CO_2_, 95% air environment at 37°C.

### Min6 cell line GSIS studies

Min6 cells at 90% confluence in 6 cm dishes were first incubated in Krebs Ring Buffer (KRB, consisting of NaCl 130 mM, KCl 4.7 mM, NaH_2_PO_4_ 0.5 mM, MgSO_4_ 1 mM, CaCl_2_ 1.5 mM, HEPES 2.4 mg/mL, BSA 1 mg/mL, pH to 7.4) without glucose for 1 hour, followed by 1 hour incubation in 2.8 mM glucose (low glucose – LG) KRB. Next, the cells were stimulated by incubation in 16.8 mM glucose (high glucose – HG) KRB for 1 hour (or in the control condition). At the end of the incubation period, incubation media viz., LG and HG KRB were collected for secreted insulin measurement. Min6 cells were also collected for total insulin measurements. Secreted insulin and total insulin were measured by Mouse Ultrasensitive Insulin ELISA (ALPCO, NH, USA).

### Min6 cell line ATP and citrate measurement

For ATP and citrate measurements, Min6 cells plated at the same cell number were treated with low or high glucose as described for GSIS at 90% confluence. At the end of the incubation period, cells were lysed for ATP (ab83355, Abcam) and citrate (ab83396, Abcam) measurements according to the manufacturer’s instructions.

### Experimental design for islet or Min6 cell studies

After an overnight recovery from the isolation procedure, the same experimental procedure was carried out as the GSIS experiment with islets (100 ~ 200 isolated islets) or Min6 cells at 90% confluence in 10 cm dishes, a batch of islets/cells was incubated in fresh LG KRB buffer. At the end of the incubation period, incubation media viz., LG and HG KRB were collected for LC-MS analyzes. Min6 cells and islets were also collected. For normalization of metabolite concentration, 1/10th of the Min6 cells in each cell suspension were lysed for protein quantification by Pierce Rapid Gold BCA Protein Assay Kit (Thermo Fisher, MA, USA). The rest was used for LC-MS metabolite quantification. For the islet studies, islet number per sample was used for normalization of metabolite concentration. For these experiments, non-labeled glucose was used to supplement KRB for islets, while U-^13^C_6_ labeled glucose was used for the Min6 cells.

### Acetate incorporation assay for Min6 cells

Min6 cells in 6-well plates at 90% confluency were first incubated in no glucose KRB for 1 hour, then changed to non-labeled LG KRB for 1 hour. The LG KRB was aspirated and replaced by fresh non-labeled LG or HG KRB buffer with 0.5µCi^14^C acetate (Perkin Elmer, MA, USA) for another hour. Cells were then lysed in 200 µL RIPA buffer (Sigma Aldrich) with Halt Protease Inhibitor (Thermo fisher). 1 µL of lysate was used to measure protein concentration by Pierce Rapid Gold BCA Protein Assay Kit (Thermo fisher). The rest of the lysate and cell debris were mixed with Ultima Gold LSC Cocktail (Sigma Aldrich) and read in the scintillation counter (Beckman Coulter LS6500, CA, USA). Readings were normalized by protein concentration of the sample.

### LC-MS sample preparation, derivatization, instrumentation, and data acquisition for Min6 cells and islets

Sodium Acetate (S2889, Sigma-Aldrich) and Sodium Pyruvate (P2256, Sigma-Aldrich) were individually dissolved in water to make a 100 mM solution. A stock solution of 2 analytes was made as a 1 mM solution with 50% Methanol (MeOH) in water. It was further diluted with 50% MeOH to obtain a 25 µM and 50 µM standard solution, which was used for Quality Control (QC). All LC-MS grade solvents were purchased from Sigma-Aldrich.

### Sample preparation

The cells (Min6 cells and islets) and collected LG and HG KRB were stored at −80°C and were thawed on ice prior to further use. For Min6 cells, 300 µL of ice-cold 50% MeOH in water was added and the cells were homogenized using high-impact Zirconium beads (1.5 mm diameter) in a Bead Bug homogenizer. For islets, 200 µL of ice-cold 50% MeOH in water was added, islets were subject to freeze-thaw cycles (5X) and finally incubated at 60°C to rupture the cell membrane. The cell lysates were centrifuged at 15,000 rpm for 10 min at 4°C and the supernatant was transferred to a clean tube for derivatization. LG and HG KRB required no further processing before derivatization.

### Standards

For preparation of internal standard/isotope labeled standards (IS) mix: 100 μL standard solution of sodium acetate and sodium pyruvate was derivatized with 2 mg 13C 3-NPH and 50 μL 120 mM EDC containing 6% pyridine in 50% MeOH. The reaction mixture was incubated for 30 min at 40°C, after which it was diluted with 100 mL 10% MeOH in water. The standard solutions and samples were mixed with stable isotope-labeled standards (IS) for LC-MS Analysis.

### Derivatization

For Min6 cells and their collected LG and HG KRB, 60 µL of the standard or sample was mixed with 30 µL of 200 mM 3-NPH in 50% aqueous MeOH and 30 µL of 120 mM EDC containing 6% pyridine, in the same solution. The reaction was allowed to proceed for 30 min at 40°C. The reaction mixture was then diluted with 10% MeOH. For islets and their collected LG and HG KRB culture media, 40 µL of each standard solution/sample supernatant was mixed with 10 µL of 3-NPH (400 mM in 50% aqueous methanol) and 10uL of EDC (240 mM with 6% pyridine in 50% methanol). The reaction was carried out for 30 min at 40°C. The reaction mixture was then diluted with 10% MeOH. The standard solutions and samples were mixed with stable isotope-labeled standards (IS) for LC/MS Analysis.

### Instrumentation and data acquisition

5 µL of the calibrator/sample was injected into an AB SCIEX 5500 QTRAP coupled with Agilent 1290 UPLC system. All samples were eluted by Agilent Poroshell column 120 EC-C18 2.7 μm, 2.1 × 100 mm (P/N 695,775–902) with a flow rate of 450 μL/min. The column compartment was kept at 45°C. LC elution started with 99% mobile phase A (0.1% FA in H2O) for 1 min, followed by a linear gradient increase of mobile phase B (0.1% FA in ACN) from 1% to 10% in 1 min, from 10% to 65% in 6 min and then to 90% in 0.1 min. Columns were washed with 90% B for 3 min then re-equilibrated back to the initial condition (99% A) for 3 min. The autosampler was maintained at 10°C. MS data were acquired by Multiple reaction monitoring(MRM)scan at negative mode. The ESI spray voltage and source temperature were kept at 4.5 kV and 450°C.

### LC-MS data analysis

The data analysis was conducted by Sciex MultiQuant software (Version 3.0.3, AB Sciex, Ltd., Framingham, MA, USA).

#### RNA isolation and gene expression

RNA was extracted either by RLT (Qiagen) from islets and Min6 cells or TRIzol reagent (Life Technologies) and chloroform using phase separation for liver and muscle. RNA was purified using the RNeasy Mini Kit (Qiagen). One microgram of RNA from liver, muscle, and Min6 cells, and 200 ng for islets was reverse transcribed using the iScript cDNA synthesis kit (Bio-Rad Laboratories) and transcribed using the iTaq Universal SYBR Green Supermix (Bio-Rad Laboratories). Gene expression was calculated using the 2^–∆∆Ct^ method against housekeeping gene, *β-actin*. Primer sequences used for amplification are listed below:

**Table:** 

Gene	Forward Primer 5’-3’	Reverse Primer 5’-3’
*Mct1*	TGTTAGTCGGAGCCTTCATTTC	CACTGGTCGTTGCACTGAATA
*Mct2*	GCTGGGTCGTAGTCTGTGC	ATCCAAGCGATCTGACTGGAG
*Mct3*	TCGTGGAGCTGTACCTGAC	CCGCTCGAAGTAGAGTCCC
*Mct4*	TCACGGGTTTCTCCTACGC	GCCAAAGCGGTTCACACAC
*β-actin*	GTTACAGGAAGTCCCTCACCC	CAGACCTGGGCCATTCAGAAA

### Statistics

Data were expressed as mean ± standard error and analyzes are done by student’s *t* test or by Two-way ANOVA with a post hoc test using GraphPad Prism 9 (GraphPad, CA, USA).

## Results

**Figure 1.**
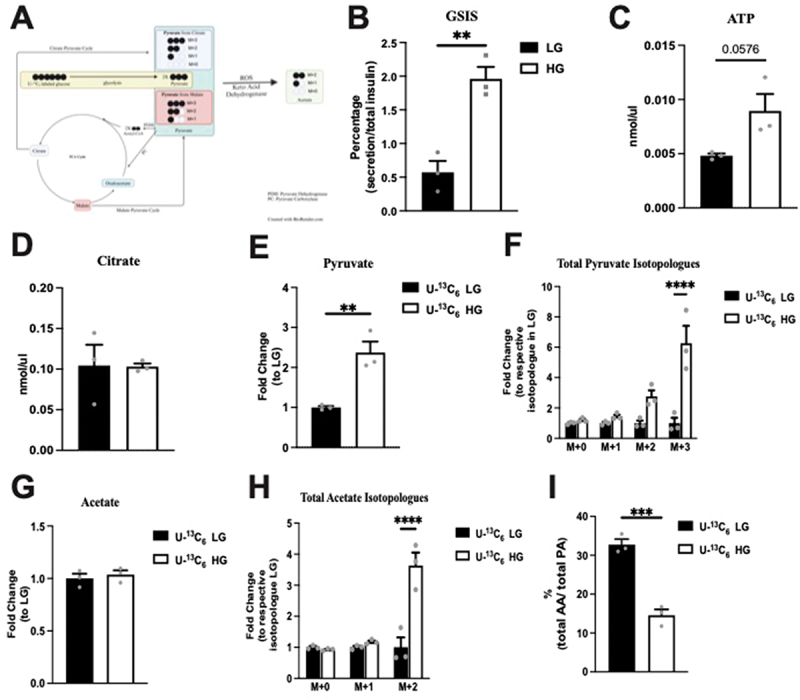
Total (intracellular + extracellular) levels of studied metabolites in response to incubation of Min6 cells under low glucose (LG, 2.8 mM) and high glucose (HG, 16.8 mM). (a) Illustration of the mechanism for labeled pyruvate and acetate produced from U-^13^C_6_ labeled glucose. GSIS studies (b), and intracellular ATP (c) and intracellular citrate (d) levels of LG and HG incubated Min6 cells. Total levels of (e) pyruvate, (f) pyruvate isotopologues, (g) acetate and (h) acetate isotopologues represented as fold change relative to U-^13^C_6_ LG condition. (i) Percentage of total acetate to total pyruvate in U-^13^C_6_ LG and HG condition. Experiments were conducted three independent times in triplicate each time. Data was analyzed by student’s *t* test (1B, 1C, 1D, 1E, 1 G and 1I) or by Two-way ANOVA (1F and 1 H). *p < .05, **p < .01, ***p < .001 and ****p < .0001. Black bar = LG, white bar = HG. Pyruvate Dehydrogenase (PDH), Pyruvate Carboxylase (PC), acetic acid (AA), and pyruvic acid (PA).

Hyperactive glucose metabolism in mammalian cells can lead to acetate production from the breakdown of pyruvate.^26^ A recent report specifically suggested that pancreatic β cells may be able to release acetate under high glucose conditions; however, concerns exist regarding the accuracy of acetate measurements using enzyme-based colorimetric assay.^20^ Using LC-MS, which is the gold standard method to measure acetate and pyruvate, we quantified the levels of these molecules in Min6 cells and their culture media (KRB) following low glucose (LG) or high glucose (HG) incubation. In these studies, we used U-^13^C_6_ labeled glucose to differentiate newly synthesized pyruvate/acetate isotopologues from preexisting amounts, with newly synthesized pyruvate/acetate containing labeled carbon. Additionally, we can also differentiate pyruvate derived from glycolysis (M + 3 pyruvate) and the TCA cycle (most in the form of M + 2 pyruvate, lesser in M + 1 pyruvate, and the least as M + 0 pyruvate), as mouse islets and pancreatic β cell lines have multiple shuttles that convert TCA cycle intermediates, specifically citrate and malate, to pyruvate (Figure 1a).^6,7^

### Acetate production is minimal and glucose independent in Min6 cells

First, to verify that our Min6 cells are glucose responsive and secrete insulin, we examined the GSIS by Min6 cells in LG and HG. HG elicited a 3.5-fold increase in insulin secretion compared to LG (Figure 1b). ATP and citrate concentrations were measured in Min6 cell lysates after incubation in LG and HG. Compared to the LG, ATP production by Min6 cells incubated was 1.9 times higher (*p* = 0.0576), as expected (Figure 1c). The citrate concentration was equivalent between the LG and HG conditions, indicating TCA as a minor contributor to pyruvate in Min6 cells (Figure 1d). Collectively, these data suggest that our Min6 cells were glucose responsive.

Next, we assessed the overall levels of pyruvate and acetate following the incubation of Min6 cells with glucose to determine the collective change in intracellular + extracellular amounts in response to glucose. As seen in Figure 1(e), compared to the LG conditions, total (intracellular + extracellular) pyruvate was significantly elevated under HG conditions, confirming the expected increase in pyruvate under high glucose levels. Only M + 3 pyruvate was significantly increased in HG condition without a significant change in M + 2 pyruvate (Figure 1f). These data and the unchanged citrate levels (Figure 1d) suggest that under the HG conditions, minimal newly synthesized pyruvate entered the TCA cycle. In contrast, total (intracellular + extracellular) acetate remained unchanged between LG and HG conditions (Figure 1g). Similar to total pyruvate, M + 2 acetate was also significantly increased in HG (Figure 1h), suggesting the presence of newly synthesized acetate from U-^13^C_6_ labeled glucose. Next, we compared the relative amount of acetate to pyruvate under LG and HG conditions. Importantly, the acetate amount was only 32.75% of the total pyruvate in LG, and this ratio decreased to 14.61% in the HG condition as there was a larger increase in pyruvate production without a proportional increase in acetate (Figure 1i). These data suggest that total acetate (intracellular + extracellular) was lower than total pyruvate (intracellular + extracellular) and was not affected by extracellular glucose levels.

### Intracellular pyruvate and acetate production is glucose dependent in Min6 cells

**Figure 2.**
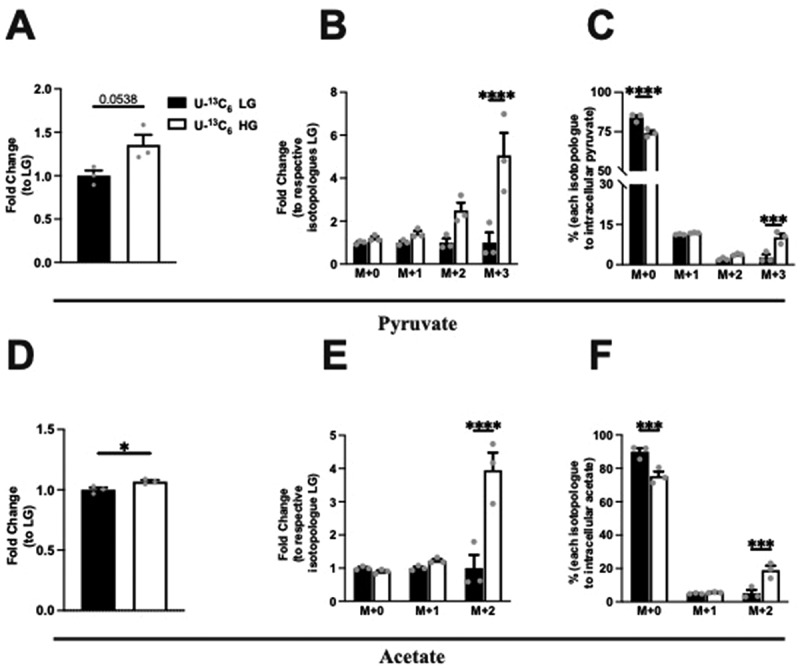
Intracellular pyruvate, acetate, and their isotopologue levels following incubation of Min6 cells in U-^13^C_6_ low glucose (U-^13^C_6_ LG, 2.8 mM) or high glucose (U-^13^C_6_ HG, 16.8 mM). (a) Total intracellular pyruvate level represented as fold change relative to U-^13^C_6_ LG condition. (b) Intracellular pyruvate isotopologues levels represented as fold change relative to its respective isotopologues levels in U-^13^C_6_ LG condition. (c) Percentage of intracellular pyruvate isotopologues within U-^13^C_6_ LG or HG condition. (d) Total intracellular acetate level represented as fold change relative to U-^13^C_6_ LG condition. (e) Intracellular acetate isotopologues levels represented as fold change relative to its respective isotopologues levels in U-^13^C_6_ LG condition. (f) Percentage of intracellular acetate isotopologues within U-^13^C_6_ LG or HG condition. Experiments were conducted three times in triplicate each time. Data was analyzed by student’s *t* test (2A and 2D) or by Two-way ANOVA (2B, 2C, 2E and 2F). *p < .05, **p < .01, ***p < .001 and ****p < .0001. Black bar = U-^13^C_6_ LG, white bar = U-^13^C_6_ HG.

Intracellular pyruvate and acetate, and their isotopologue levels were next assessed following the incubation of Min6 cells in U-^13^C_6_ LG and HG. First, under HG conditions as compared to LG, intracellular pyruvate increased, although not significantly (Figure 2a). When examining the fold change of the isotopologue amounts, a significant fold increase (relative to the respective isotopologue amount in LG) occurred in M + 3 pyruvate in HG condition (Figure 2b). Next, examining the percentages within the LG or HG conditions, the isotopologue percentages significantly increased in M + 3 in HG, compared to the proportional percentages in LG, with a corresponding drop in M + 0 (Figure 2c).

Examining the intracellular acetate changes, a small but significant increase (6.8%, *p* = 0.035) occurred in HG condition (Figure 2d). When examining the fold change of the isotopologue amounts, the fold increase (relative to the respective isotopologue amount in LG) was significant in the M + 2 acetate in HG condition (Figure 2e). As observed with labeled pyruvate species, the isotopologue percentages of acetate increased significantly in M + 2 in HG, compared to the proportional percentage in LG, with a corresponding drop in M + 0 (Figure 2f). Taken together, these data indicate that most intracellular pyruvate and acetate were generated from unlabeled glucose, but in the HG condition with expected uptake of the added labeled glucose, more labeled pyruvate and acetate were generated.

### Glucose concentration governs extracellular pyruvate, but not acetate levels in Min6 cells

**Figure 3.**
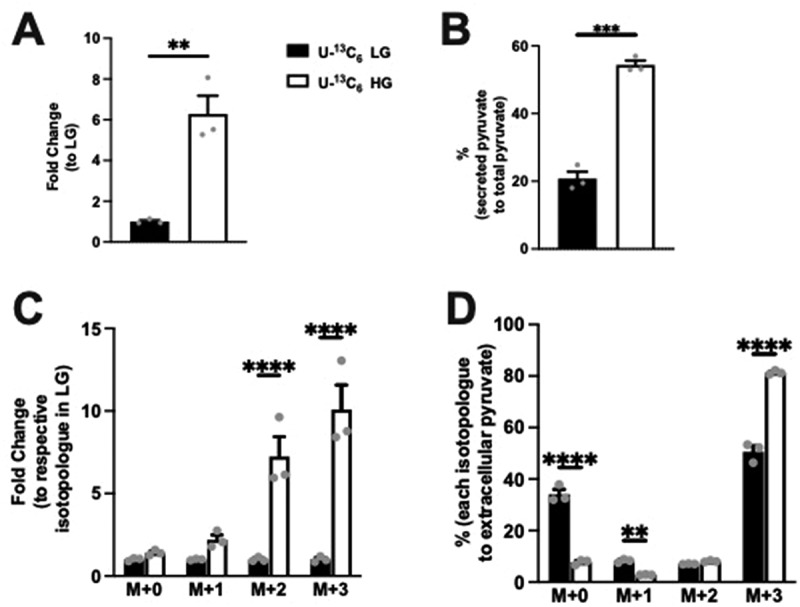
Extracellular pyruvate and levels of its isotopologues following incubation of Min6 cells in U-^13^C_6_ low glucose (LG, 2.8 mM) or high glucose (HG, 16.8 mM). (a) Total extracellular pyruvate level represented as fold change relative to the U-13C_6_ LG condition. (b) Percentage extracellular pyruvate to total (intracellular + extracellular) pyruvate in U-^13^C_6_ LG and HG condition. (c) Extracellular pyruvate isotopologues levels represented as fold change relative to its respective isotopologues levels in U-^13^C_6_ LG condition. (d) Percentage of extracellular pyruvate isotopologues within U-^13^C_6_ LG or HG condition. Experiments were conducted three times in triplicate each time. Data was analyzed by student’s *t* test (3A and 3B) or by Two-way ANOVA (3C and 3D). *p < .05, **p < .01, ***p < .001 and ****p < .0001. Black bar = U-^13^C_6_ LG, white bar = U-^13^C_6_ HG.

To determine whether glucose affects extracellular pyruvate and acetate levels, extracellular pyruvate and acetate concentrations were measured. Specifically, KRB cultured with Min6 cells at LG or HG (using U-^13^C_6_ stable glucose isotopes) were quantified by LC-MS. Interestingly, we observed a significant fold increase in extracellular pyruvate in HG condition compared to LG (Figure 3a). The extracellular pyruvate comprised 20.76% and 54.37% of the overall pyruvate production in LG and HG, respectively (Figure 3b). Examining the fold change of the isotopologues levels, the increase in extracellular pyruvate in HG condition was primarily noted in the M + 2 and M + 3 groups (Figure 3c). Next, when we examined the percentage change within each group, we observed the M + 0 and M + 1%s to be significantly reduced (Figure 3d) with the M + 3 pyruvate being the most abundant isotopologue (Figure 3d) in the HG condition. These data indicate that most of the extracellular pyruvate was derived from labeled glucose in both LG and HG conditions.

**Figure 4.**
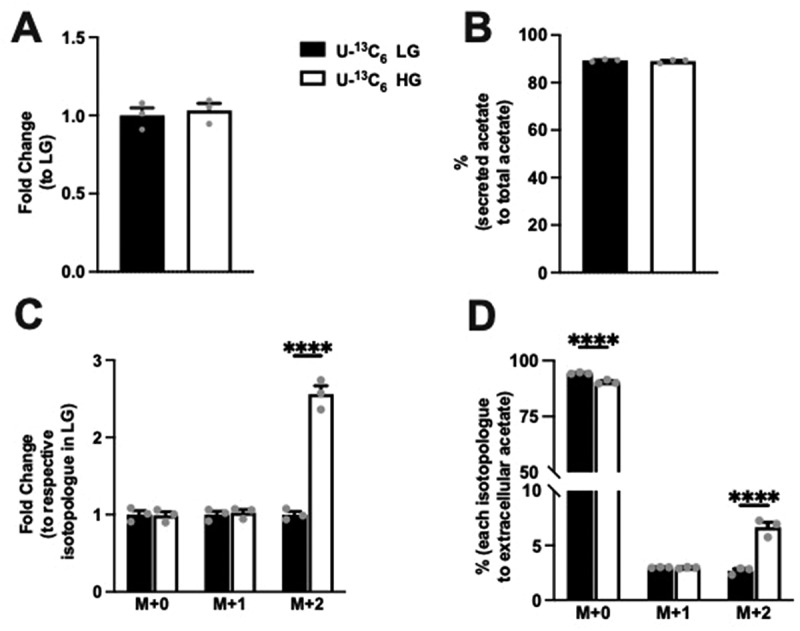
Extracellular acetate and levels of its isotopologues following incubation of Min6 cells in U-^13^C_6_ LG and U-^13^C_6_ HG. (a) Total extracellular acetate level represented as fold change relative to U-^13^C_6_ LG condition. (b) Percentage of extracellular acetate to total (intracellular + extracellular) acetate in U-^13^C_6_ LG and HG condition. (c) Extracellular acetate isotopologues levels represented as fold change relative to its respective isotopologues levels in U-^13^C_6_ LG condition. (d) Percentage of extracellular acetate isotopologues in U-^13^C_6_ LG and HG condition. Experiments were conducted three times in triplicate each time. Data was analyzed by student’s *t* test (4A and 4B) or by Two-way ANOVA (4C and 4D). *p < .05, **p < .01, ***p < .001 and ****p < .0001. Black bar = U-^13^C_6_ LG, white bar = U-^13^C_6_ HG.

Examining extracellular acetate, no difference in the level was noted between LG and HG condition (Figure 4a). Interestingly, the extracellular acetate comprised 89% of overall acetate produced in both LG and HG and was not different between conditions (Figure 4b). Considering the total acetate production is a small proportion of the pyruvate produced (Figure 1c), these data demonstrate that extracellular acetate amounts were low compared to pyruvate. Examining the fold change in isotopologue levels for acetate, the fold change with isotopologue groups was the greatest in M + 2 acetate (Figure 4c,d). The percentage elevation with HG was seen primarily in the M + 2 condition. In contrast to extracellular pyruvate, M + 0 acetate was the major form regardless of glucose concentration (Figure 4d). These data indicate that M + 0 acetate is the major isotopologue of the extracellular acetate, which comes from unlabeled glucose. However, the addition of glucose did contribute to the extracellular acetate.

### Acetate incorporation in Min6 cells is not glucose dependent

**Figure 5.**
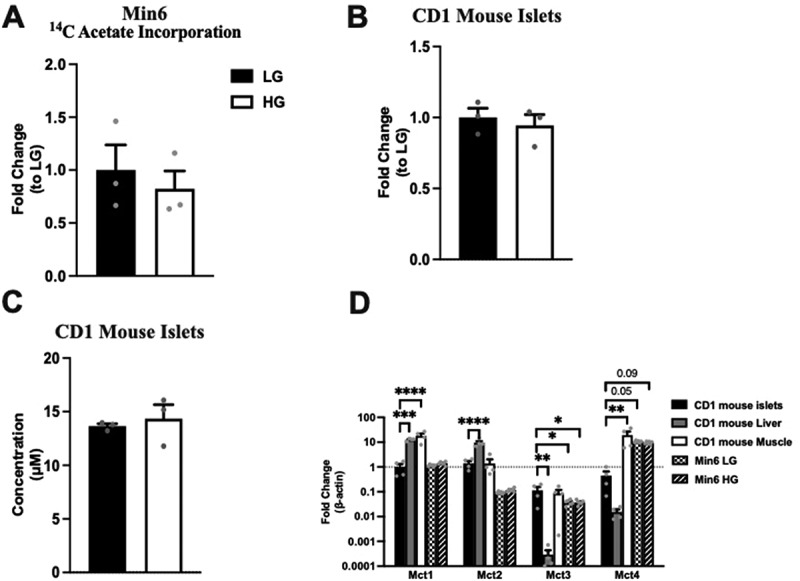
Glucose concentration does not affect extracellular acetate incorporation into Min6 cells or intracellular/extracellular acetate levels in CD1 islets. (a) ^14^C acetate incorporation level of Min6 cells represented as fold change relative to LG condition. (b) Intracellular acetate of CD1 mouse islets represented as fold change relative to LG condition. (c) Concentration (µM) of extracellular acetate CD1 mouse islets. (d) Fold change of MCT1–4 expression in CD1 mice islets, liver, muscle, LG incubated Min6 cells, or HG incubated Min6 cells relative to CD islet MCT1 expression. For 5A, experiment was conducted three times in triplicate each time. For 5B and 5C, *n* = 3 samples, each sample has 100 ~ 200 islets. For 5D, CD1 islets, liver, and muscle, the *n* = 4, and for min6 LG and min6 HG, the *n* = 6. Data was analyzed by student’s *t* test (5A, 5B and 5C) and One-way ANOVA (5D), *p < .05, **p < .01, ***p < .001 and ****p < .0001. Black bar = LG, white bar = HG.

Next, we assessed the effect of glucose concentration on acetate incorporation into the Min6 cells. As acetate transport could be bi-directional, ^14^C acetate was added into LG and HG (non-labeled glucose) KRB, and these Min6 cells were cultured for 1 h and harvested. Liquid scintillation counting of lysates to measure acetate incorporation revealed similar radioactive counts at LG and HG concentrations (Figure 5a), indicating that acetate influx in Min6 cells was independent of extracellular glucose concentration.

### Acetate secretion is not glucose dependent in CD1 mouse islets

To confirm the observation that glucose does not govern extracellular acetate levels from Min6 cells in islets, intracellular and extracellular acetate were quantified using non-labeled glucose in the same experimental setting with islets (100 ~ 200 islets per sample). LC-MS measurements showed no differences in either intracellular or extracellular acetate levels (Figure 5b,c). We have also examined the expression of MCT1–4, the transporters for short-chain monocarboxylates, in multiple tissues of CD1 mice and Min6 cells under LG and HG conditions (Figure 5d). As previously suggested, CD1 mouse islets express MCT1–4 scarcely. Min6 cells express higher levels of MCT4 than CD1 mouse islets. As compared to the islets, the liver has higher expression of MCT1 and MCT2, and the muscle has higher MCT1 and MCT4 expression. These data, in conjunction with the data obtained from Min6 cells, indicate that changes in glucose levels do not govern extracellular acetate levels.

## Discussion

Pancreatic β cells are key players in the control of blood glucose and the development of diabetes^1,2^. Recent research has suggested that SCFAs sensing receptors can regulate β-cell functions, such as insulin secretion, and can be potential therapeutic targets for diabetes^18,19,21,27^. Although many aspects of β-cell SCFA receptor functions remain unclear, the role of SCFAs in regulating insulin secretion is well established.

The current understanding of serum acetate, the primary SCFAs in serum, is predominantly derived from the gut microbiome.^28^ Multiple studies have demonstrated that acetate at millimolar range, which is considerably above the EC_50_ values for mouse FFA2 (EC_50_ = 281 uM) and FFA3 (EC_50_ = 186 uM), regulates insulin secretion.^19,20,29–31^ However, local production of acetate by different cell types during glucose metabolism is possible.^26^ It has been recently reported that pancreatic β cells release acetate in a glucose-dependent manner,^20^ and that this acetate acts in an autocrine manner to mediate insulin secretion. As this would suggest a novel mechanism to control insulin secretion, further studies are required to examine these findings.

In our study, we quantified the release of pyruvate and acetate from the β cells in the presence of different concentrations of glucose. We used LC-MS, the gold standard approach, to measure the total production of intracellular and extracellular pyruvate in Min6 cells incubated with U-^13^C_6_ labeled LG and HG. Incubation with HG led to a significant elevation of total pyruvate production. In contrast, the total acetate production was not altered. We observed an increase in intracellular pyruvate and acetate levels in HG. In comparison with LG, extracellular pyruvate was upregulated 6.2 times in HG condition, whereas extracellular acetate showed no difference. This observation was confirmed in CD1 mouse islets. The extracellular acetate concentration from the CD1 mouse islet experiments ranged from 10 µM to 20 µM (Figure 5c), which is considerably lower than the reported EC_50_ values of mouse FFA2 and FFA3.^31^ Thus, it is unlikely that acetate at this concentration is able to regulate insulin secretion.

Monocarboxylate acids, such as acetate, pyruvate, and lactate are transported across the plasma membrane by the transporters MCT1–4 to participate directly in energy metabolism in mammalian cells.^32^ In the muscle, MCT1 and MCT4 expressed in oxidative and glycolytic fibers participate in lactic acid production and partial recycling^33^. Gut microbiome-derived acetate, transported across the plasma membrane via MCTs, also contributes to muscle oxidative metabolism during exercise.^34,35^ The liver primarily expresses MCT1 that allows influx and utilization of lactate produced by the muscle as a substrate for gluconeogenesis.^36^ During prolonged fasting, MCT1 expression is increased in the hepatocytes ensuring efflux of fasting induced excess acetate and ketone bodies out of the hepatocytes.^37,38^ In the colon, acetate produced by the gut microbiome serves as one of the energy source for the colonocytes.^39^ Other organs, such as the heart and kidneys, can also utilize acetate and lactate.^36,40^ However, pancreatic β cells, due to their unique role in the control of postprandial-blood glucose, suppress the expression of genes (disallowed genes) that can impede normal β-cell insulin secretion where MCTs are among them.^22^ Only exception to this is a genetic disorder called exercise-induced hyperinsulinism, where a mutation in the *Mct1* gene promoter results in failure to suppress *Mct1* expression in pancreatic β cells, which increases the influx of pyruvate, lactate, and acetate.^41^ Because of this expression of *Mct1*, these molecules are directly channeled into the energy metabolism, which increases insulin secretion. Though Min6 cells express trace amounts of MCT4,^22^ the^14^C acetate incorporation experiment (Figure 5a) confirmed no acetate influx in Min6 cells. Thus, our data are consistent with the earlier reports discussed above.

Our study, however, has few limitations. One is that we primarily derive our conclusions from one beta cell line, the Min6 cells. We used this cell line as it is glucose responsive (Figure 1(b), though not as high as the mouse islets),^42^ where this diminished insulin secretion may be a reflection of the lack of change in citrate concentration and total (intracellular plus extracellular) M + 1 and M + 2 pyruvate amounts between LG and HG. We corroborate these data with a study on mouse islets (Figure 5). According to other metabolomics studies using primary rodent islets, TCA intermediates are significantly increased in HG, which potentially affects intracellular pyruvate and acetate concentrations.^43^ However, we did not observe any change in extracellular acetate in mouse islets between LG and HG, which we suggest is because of the low expression of MCTs in mouse islets.^44,45^ Another limitation is that we did not explore acetate secretion at different glucose concentrations with human islets. Though conducting similar studies on human islets will be important, the results could be confounding due to the known functional heterogeneity of the human islets.^42^ While the EC_50_ of human FFA2 for acetate is slightly lower than that of mouse FFA2, we do not expect this could impact insulin secretion from any local acetate released from human islets,^46^ but this warrants exploration. Nonetheless, according to several single-cell RNA-seq experiments, MCT1–4 are not expressed in human pancreatic β cells.^44,45,47,48^

The data presented in this study, obtained using appropriate detection tools, demonstrate that any significant amount of acetate is not released from mouse pancreatic β cells in response to changes in extracellular glucose concentrations. This observation is consistent with the existing data on the low expression of MCTs in the β cells. Thus, we conclude that acetate autocrine signaling through SCFAs receptors on islet β-cells is improbable.

## Supplementary Material

graphic abstract.png
