# Supplementary figures and images for: β cell acetate production and release are negligible
Source: Islets. 2024 Apr 12;16(1):2339558. doi: 10.1080/19382014.2024.2339558 (PMC11018053; doi:10.1080/19382014.2024.2339558)

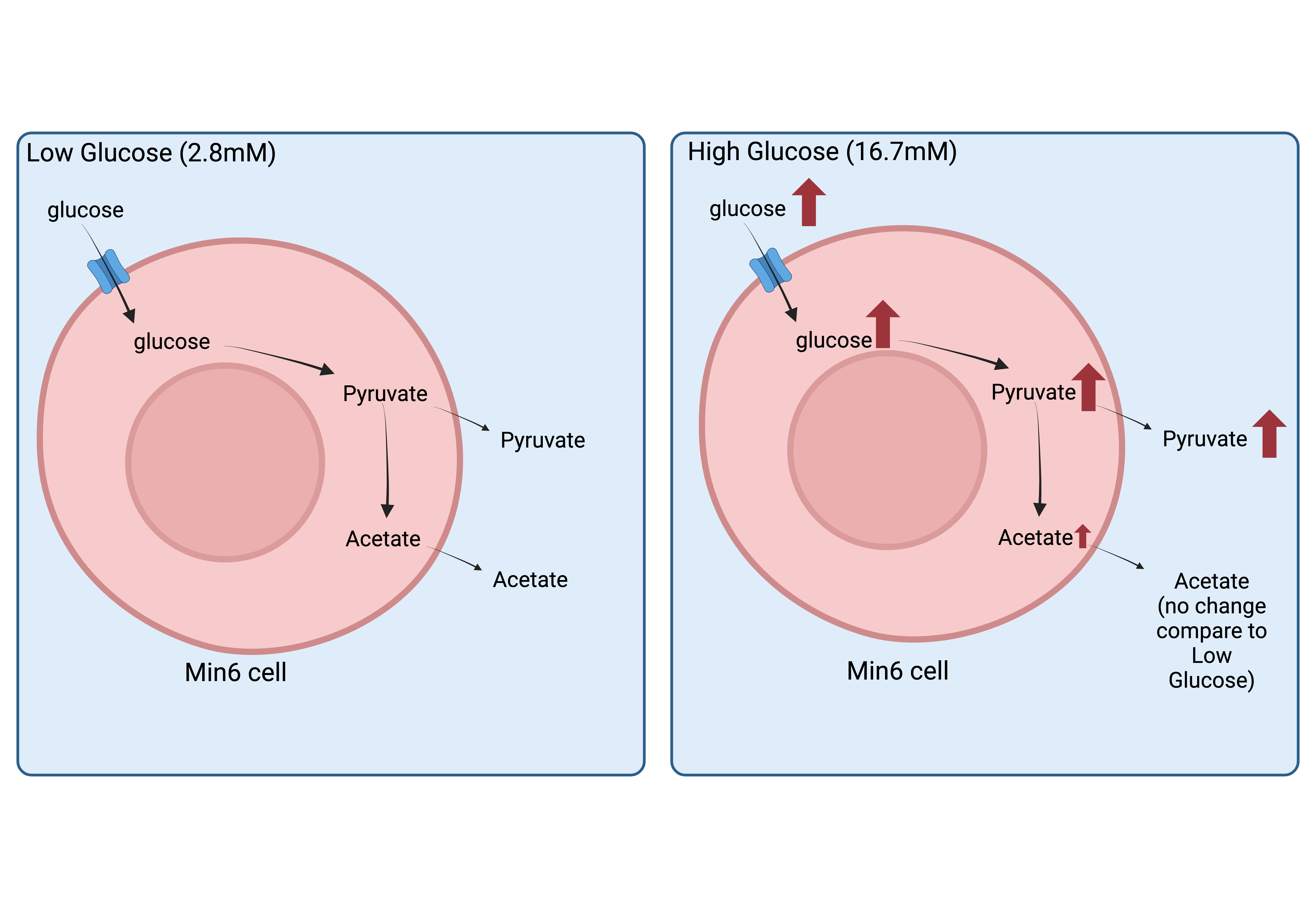

Supplement: graphic abstract.png [file KISL_A_2339558_SM5196.png]
